# Inhibition of Porcine Viruses by Different Cell-Targeted Antiviral Drugs

**DOI:** 10.3389/fmicb.2019.01853

**Published:** 2019-08-14

**Authors:** Patricia de León, María José Bustos, Elisa Torres, Rodrigo Cañas-Arranz, Francisco Sobrino, Angel L. Carrascosa

**Affiliations:** Centro de Biología Molecular “Severo Ochoa” (CSIC-UAM), Universidad Autónoma de Madrid, Madrid, Spain

**Keywords:** virus, porcine, antiviral, lauryl gallate, valproic acid, cerulenin

## Abstract

Antiviral compounds targeting cellular metabolism instead of virus components have become an interesting issue for preventing and controlling the spread of virus infection, either as sole treatment or as a complement of vaccination. Some of these compounds are involved in the control of lipid metabolism and/or membrane rearrangements. Here, we describe the effect of three of these cell-targeting antivirals: lauryl gallate (LG), valproic acid (VPA), and cerulenin (CRL) in the multiplication of viruses causing important porcine diseases. The results confirm the antiviral action in cultured cells of LG against African swine fever virus (ASFV), foot and mouth disease virus (FMDV), vesicular stomatitis virus (VSV), and swine vesicular disease virus (SVDV), as well as the inhibitory effect of VPA and CRL on ASFV infection. Other gallate esters have been also assayed for their inhibition of FMDV growth. The combined action of these antivirals has been also tested in ASFV infections, with some synergistic effects when LG and VPA were co-administered. Regarding the mode of action of the antivirals, experiments on the effect of the time of its addition in infected cell cultures indicated that the inhibition by VPA and CRL occurred at early times after ASFV infection, while LG inhibited a late step in FMDV infection. In all the cases, the presence of the antiviral reduced or abolished the induction of virus-specific proteins. Interestingly, LG also reduced mortality and FMDV load in a mouse model. The possible use of cell-targeted antivirals against porcine diseases is discussed.

## Introduction

Conventional antiviral drugs (AVs) that interfere with viral proteins or functions have been developed against a number of different animal viral diseases, but these compounds often lead to selection of drug resistance in virus populations evolving under selective pressures (Cuypers et al., 2016; Maldonado and Mansky, 2018). An alternative approach is the identification of compounds targeted against cellular functions required for viruses to complete their multiplication cycle. Hence, an efficient non-conventional cell-targeted antiviral would inhibit viral entry and/or replication at a non-toxic concentration for the cell and, ideally, it would help to control a wide variety of viral diseases and virus strains. Following this approach, we have focused our studies on three compounds: lauryl gallate (LG), valproic acid (VPA), and cerulenin (CRL) (Table 1) that can interfere with cell metabolism and have been previously described as potential AVs.

**Table 1:** Formula and cytotoxicity of antivirals in different cell lines. 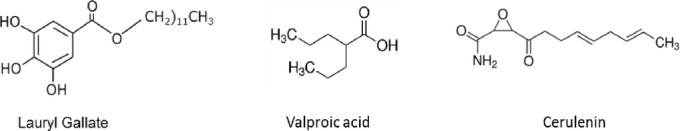

	Antiviral^a^		
Cell line	LG	VPA	CRL
Vero	45 μM	50 mM	45 μM
COS	33 μM	180 mM	60 μM
BHK	75 μM	175 mM	60 μM
IBRS-2	30 μM		40 μM

a *Data expressed as CC_50_, concentration of antiviral yielding a 50% of cell viability in MTT assay*.

LG is an ester derivative of gallic acid, a natural plant phenolic compound. It has been widely used as antioxidant (E312) in food manufacturing, as well as in the pharmaceutical and cosmetic industries. LG protects cells from oxidative stress, in a process associated with radical scavenging, inhibition of enzymes involved in lipid peroxidation, and increasing expression of antioxidant genes (Kubo et al., 2002). Due to its hydrophobic properties, LG can disrupt biomembranes and cause protein inactivation (Jurak and Minones, 2016). This interaction with lipid molecules can be correlated with different pharmacological effects against bacterial cells and viruses (Ogita et al., 2004; Hurtado et al., 2008). We have previously demonstrated that viral production was strongly inhibited at non-toxic concentrations of LG, in different cell lines infected with African swine fever virus (ASFV), herpes simplex virus (HSV-1), vaccinia virus (VV), influenza virus, transmissible gastroenteritis virus and Sindbis virus (Hurtado et al., 2008).

VPA is a branched short-chain fatty acid that has been widely used for treatment of neurological diseases such as epilepsy, migraines, or bipolar disorders (Terbach and Williams, 2009). VPA has been shown to alter a variety of signaling pathways, including an increase in GABA neurotransmission, inhibition of histone deacetylases (HDACs), inhibition of glycogen synthase kinase beta (GSK3β), or attenuation of phospholipid signaling (Terbach and Williams, 2009). Therapeutic roles of VPA have been proposed in cancer, Alzheimer’s disease, and HIV treatment (Monti et al., 2010). We previously showed that VPA is a potent inhibitor of the multiplication of several enveloped viruses (Vazquez-Calvo et al., 2011). VPA treatment of vesicular stomatitis virus (VSV)-infected cells reduced viral production without significantly affecting viral RNA and protein synthesis but interfering with both cell release and specific infectivity of virion particles, while VPA blocked virus entry and multiplication during West Nile virus (WNV) (Vazquez-Calvo et al., 2013) and HSV-1 infection (Crespillo et al., 2016).

The FASN synthase inhibitor cerulenin has been shown to reduce production of several viruses such as VV, WNV, and Semliki Forest virus (Martin-Acebes et al., 2011; Greseth and Traktman, 2014; Royle et al., 2017), suggesting that *de novo* fatty acid biosynthesis is relevant for numerous viral infections.

Despite the considerable effort invested in new vaccine designs, viruses still produce many porcine diseases with economic importance, such as those caused by ASFV, foot and mouth disease virus (FMDV), VSV, or swine vesicular disease virus (SVDV), and many aspects regarding their spread and persistence remain elusive. ASFV causes an important animal disease for which no effective vaccine or therapeutic treatment is available (Sanchez-Cordon et al., 2018). Likewise, FMD is the animal viral disease with the largest economic impact worldwide. Conventional vaccines have allowed FMD control and eventual eradication of the disease when properly produced and administered, but pose shortcomings such as the need of high-containment facilities for production, short-lived immunity, and short shelf-life after formulation (Sobrino et al., 2001). A limited repertoire of antiviral drugs is available for ASFV and FMDV and those targeting cell host factors are extracts of several plants, marine microalgae, stilbenes, and other organisms (Fabregas et al., 1999; Galindo et al., 2011; Mottola et al., 2013; Hakobyan et al., 2016; De Vleeschauwer et al., 2017). There is a current trend to consider antiviral compounds as a valuable complement to vaccination for the efficient short-term control of animal viruses such as FMDV.

## Materials and Methods

### Cells and Viruses

Vero and COS (both from African green monkey kidney), BHK21 (baby hamster kidney), and IBRS-2 (pig kidney) cells were grown at 37°C in DMEM medium supplemented with 5 or 10% fetal calf serum. ASFV isolates, either from a field strain (E70) or a Vero cell-adapted strain (Ba71V), were grown in swine alveolar macrophages (E70) or in Vero cells and titrated on COS cell monolayers, as described (Carrascosa et al., 2011). Type C FMDV C-S8 isolate (Sobrino et al., 1983) and VSV Indiana (Novella et al., 1996) virus stocks were grown in BHK21 cells, while type O FMDV 0-UKG 11/2001 (The Pirbright Institute, UK) and SVDV SP93 (Borrego et al., 2002) viral stocks were produced in IBRS-2 cells.

### Virus Infection and Antiviral Treatment

Cell monolayers were grown on multiwell plates and incubated for 1 h with different AVs (purchased from Sigma). Stock solutions of LG, VPA, and CRL were prepared in DMEM (VPA) or 2% ethanol (a concentration previously shown to preserve more than 80% cell viability) and diluted in DMEM to prepare the working stocks. After 1 h of incubation with AVs, duplicate cultures were infected with the corresponding virus in a reduced volume of medium containing the AV, for 1–2 h; the virus inoculum removed and cells washed twice with medium, before the addition of drug-containing fresh medium (supplemented with 2% FCS). Cultures were then incubated until massive cytopathic effect was observed. Total virus (intracellular and extracellular) production was evaluated by plaque assay on the corresponding virus-sensitive cells. From the dose-effect relationship of each AV on each virus yield, 50% inhibitory concentrations (IC_50_) were determined.

### Cell Viability Assay

**Figure 1:**
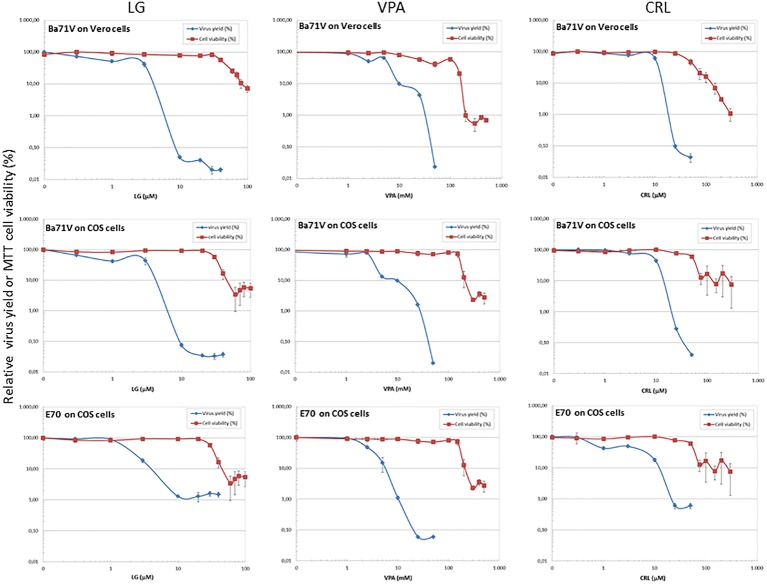
Inhibition of ASFV production by different AVs. Vero or COS cell monolayers were incubated for 1 h with LG (0–40 μM), VPA (0–50 mM), or CRL (0–50 μM) and then infected with ASFV Ba71V or E70 strains, in medium containing the same AV concentration. Total virus produced at 24 h.p.i. was titrated by plaque assay on Vero or COS cell monolayers in duplicate samples and represented as virus yield (%). The viability of non-infected Vero or COS cells incubated in the same conditions, assayed by MTT, is also represented as cell viability (%).

**Figure 2:**
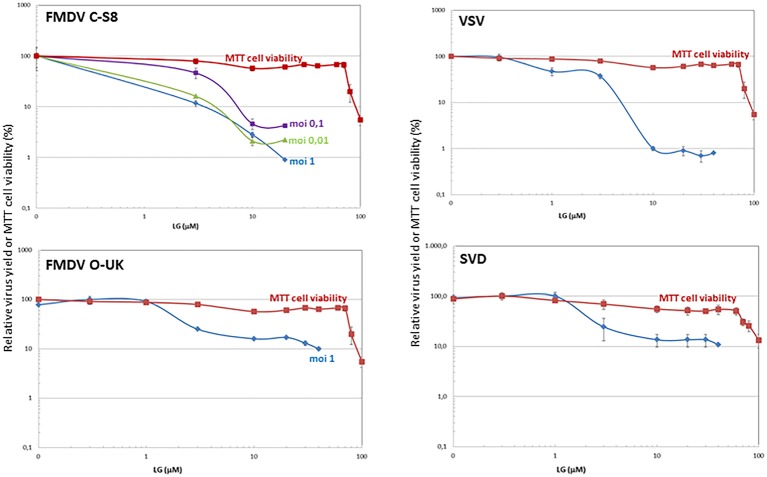
Inhibition of vesicular porcine viruses production by LG. BHK21 or IBRS-2 cell monolayers were incubated for 1 h with LG (0–40 μM) and then infected with FMDV C-S8 or O-UK isolates, VSV Indiana strain or SVDV SP93 isolate in medium containing the same LG concentrations. Total virus produced at 24 h.p.i. was titrated by plaque assay on BHK21 (for FMDV C-S8 and VSV) or IBRS-2 (for FMDV O-UK and SVDV) cell monolayers in duplicate samples, and represented as relative virus yield (%). The viability of non-infected BHK21 or IBRS-2 cells incubated in the same conditions, assayed by MTT, is also represented as cell viability (%).

Cell viability determinations were performed by the MTT assay, as described (Hurtado et al., 2008). Briefly, cell monolayers were grown on 96-well plates before the addition of the AV (six wells for each dose). After 24 h of incubation, MTT-containing culture medium was added and cells were incubated for 2 h and lysed with SDS-containing lysis solution. Absorbance at 550 nm was determined after 15 min of incubation, and average values (from six wells) obtained were subtracted from the background levels (in the absence of cells) and compared with the data scored in the absence of drug (100% viability). CC_50_ values (concentration of AV yielding a 50% of cell viability) shown in Table 1 were determined from the viability curves of each cell line with each AV (Figures 1, 2).

### Direct Virucidal Effect

**Figure 3:**
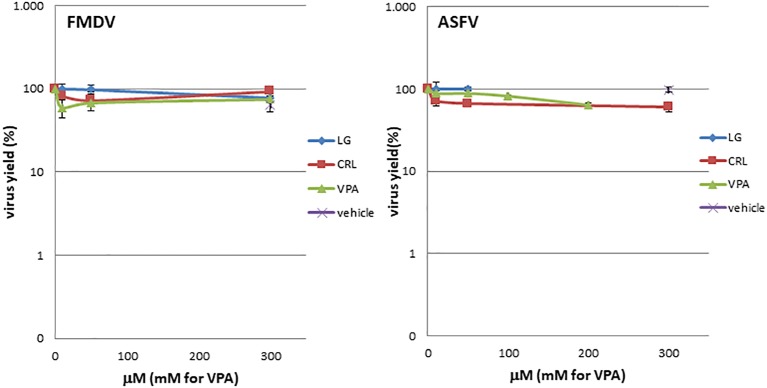
LG, CRL, and VPA virotoxicity on FMDV or ASFV. Virus suspensions were incubated for 1 h at room temperature with LG, VPA, or CRL (from 0 to 300 μM for LG and CRL, or 0 to 300 mM for VPA); controls with viruses incubated in 2% ethanol in the absence of the drug were included (vehicle). At each AV concentration, total virus was titrated by plaque assay.

To determine a possible direct virucidal effect of the AVs on virus particles, suspensions of either ASFV or FMDV (10^5^ pfu) in 0.9 ml of culture medium were incubated with 0.1 ml of LG, VPA, or CRL solutions (from 0 to 300 μM for LG and CRL, or 0 to 300 mM for VPA); controls with viruses incubated in 2% ethanol in the absence of the drug were included. After 1 h of incubation at room temperature, samples were diluted (1:1000) and titrated by plaque assay on virus-sensitive cell monolayers (COS for ASFV and BHK for FMDV samples). Virus titers were compared in duplicate samples with those obtained in virus samples incubated in the absence of the drug (Figure 3).

### Time of Addition Assay

To address the step in the virus cycle targeted by each AV, susceptible cell monolayers (Vero for ASFV or BHK and IBRS2 for FMDV) were infected with virus at a M.O.I. of 2 (ASFV) or 0.1, 1, or 5 (FMDV) pfu/cell. After adsorption, the inoculum was removed and cells were further incubated. At different times (from 3 h before infection to 24 h after infection), AVs were added to duplicate wells at the selected inhibitory concentration (10 μM LG, 20 mM VPA, or 25 μM CRL), and the cultures were further incubated until 24 h post-infection (h.p.i.), collected, and the total virus productivity evaluated by plaque assay.

### Virus-Specific Protein Expression

Vero cells were pre-treated before virus infection with 20 mM VPA or 25 μM CRL for 1 h and then infected with the ASFV isolate Ba71V (M.O.I. of 2 pfu/cell) in the presence of the AV. Virus inoculum was removed and cells were further incubated with the AV in fresh medium. At 0, 4, and 24 h.p.i., cell cultures were collected and lysed in TNT buffer (20 mM Tris–HCl pH 7.5, 0.2 M NaCl, 1% Triton X100) supplemented with protease inhibitor cocktail tablets (Roche). Total intracellular proteins (20–30 μg) were separated by 12% polyacrylamide gel electrophoresis and electroblotted. After incubation with rabbit antiserum specific for ASFV proteins induced late in the infection, membranes were exposed to horseradish peroxidase-conjugated antirabbit antibody (Amersham) and revealed by ECL autoradiography.

In the case of FMDV-specific proteins, BHK21 cells were pre-treated with 10 μM LG for 1 h and infected with C-S8 isolate (M.O.I. of 1 pfu/cell) in the presence of 10 μM LG as indicated above. Cell extracts were collected and the blotted proteins incubated with MABs SD6 or 2D2 (specific for VP1 and 3C FMDV proteins, respectively) (Garcia-Briones et al., 2006), and revealed as above.

### Extraction of Foot and Mouth Disease Virus RNA and RT-qPCR

BHK21 cells were pre-treated with LG for 1 h and infected with FMDV C-S8 isolate (M.O.I. of 1 pfu/cell) in the presence of 10 μM LG. Virus inoculum was removed and infected cultures were further incubated with LG in fresh medium. At 0, 1, 2, 3, and 8 h.p.i, cultures were collected and total (intracellular and extracellular) RNA was extracted using Tri-Reagent (Sigma). cDNA was synthesized using SuperScript III reverse transcriptase (Invitrogen), and qPCR was performed using LightCycler FastStart DNA Master SYBR Green I (Roche) containing 10 pmol each of forward and reverse primers that amplified a conserved 290-bp region in the 3D gene (Saiz et al., 2003). The qPCR was performed on a LightCycler 2.0 instrument. The number of FMDV RNA copies (VRC) was inferred from a standard curve prepared using 10-fold dilutions of cDNA from plasmid PMT28 encoding the genomic FMDV RNA (Garcia-Arriaza et al., 2004).

### “*In vivo*” Model for Foot and Mouth Disease Virus Inhibition by Lauryl Gallate

LG was initially dissolved in DMSO and then diluted in Tween80: ethanol: saline (1:1:8). Groups of 6–12 8-week-old C57 (B16J) female mice were inoculated intraperitoneally (i.p.) as described (Baranowski et al., 2003) with different concentrations of LG and administration schedules. Mice were challenged i.p. with 10^3^ or 10^4^ pfu/mouse of type C FMDV C-S8 and monitored daily for signs of infection up to 4 days p.i. Control mice were inoculated with the same volume of PBS alone plus vehicle. Mice exhibiting clear signs of disease, as well as all surviving mice at the end of the experiment, were anesthetized and euthanized.

To determine viral load after LG treatment, three animals per group were bled and euthanized at day 2 p.i. for further RNA extraction from blood, spleen, lung, heart, and thymus samples, using Tri-Reagent (Sigma) and RT-qPCR was performed as described above.

### Statistical Analysis

Data handling and analysis were performed using Graph Prism 6.01 software. Statistical differences were determined using a Student’s *t* test (*p* < 0.05).

## Results and Discussion

### Direct Virotoxicity of Antiviral Drugs on African Swine Fever Virus and Foot and Mouth Disease Virus

Direct virucidal effect of the AVs selected for this study in ASFV or FMDV infections was assayed by incubation of virus samples for 1 h at room temperature with increasing concentrations (from 0 to 300 μM for LG and CRL, or 0 to 300 mM for VPA) of the drugs. As shown in Figure 3, no direct toxicity was observed in ASFV or FMDV samples when incubated with the compounds in the range of concentrations analyzed.

### Inhibition of African Swine Fever Virus by Different Antiviral Drugs

We assessed the inhibitory effect of VPA and CRL on ASFV infection. ASFV inhibitor LG (Hurtado et al., 2008) was included in these experiments for comparative purposes. We first assayed the cytotoxicity of LG, VPA, and CRL in different cell lines. Cultures of Vero, COS, BHK21, or IBRS-2 cells were incubated for 24 h in the presence of increasing concentrations of the AVs and the CC_50_ values were determined (Table 1). The concentrations selected to determine the potential inhibition exerted by these compounds were: 1–20 μM LG, 5–30 mM VPA, and 10–50 μM CRL. Virus yield was reduced about 3 log.u. when Vero (IC_50_: 1 μM) or COS (IC_50_: 0.4 μM) cell monolayers were infected with the non-pathogenic ASFV isolate Ba71V in the presence of LG (Figure 1), as expected from previous results (Hurtado et al., 2008). A similar reduction of ASFV production was observed in the presence of non-toxic concentrations of VPA (IC_50_: 2.5–3 mM), and of CRL (IC_50_: 9 μM). Moreover, the virulent ASFV isolate E70 was also inhibited in COS cells infected in the presence of similar concentrations of the three drugs (IC_50_: 2 μM for LG, 2.5 mM for VPA, and 3 μM for CRL).

**Figure 4:**
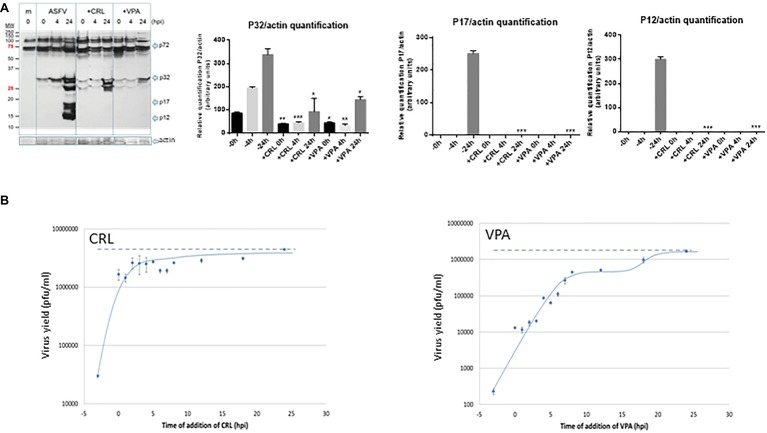
**(A)** Effect of VPA and CRL on ASFV protein expression. Vero cells were preincubated or not with 20 mM VPA or 25 μM CRL and infected with ASFV Ba71V strain at a M.O.I of 2 pfu/cell. Non-adsorbed virus was washed away and the cultures maintained in the presence or absence of the AV to complete the virus cycle. Cell extracts obtained at different h.p.i. were subjected to western blot analysis to detect early (P32) or late (P17 and P12) ASFV-induced proteins in the presence or absence of the AV. ASFV-induced proteins are indicated by arrows. Molecular weight markers are shown (MW) in the left lane. Graphs show the relative quantification of bands corresponding to P32, P17, and P12 proteins versus actin expression control. Statistically significant differences between control and AV-treated cells are indicated by asterisks (Student’s *t-*test, *p < 0.05, **p < 0.01, ***p < 0.001). **(B)** Effect of time of addition of VPA or CRL on their inhibitory activity. Vero cells were infected with ASFV Ba71V strain at a M.O.I. of 2 pfu/cell, and 20 mM VPA or 25 μM CRL was added at different times before or after infection, maintaining the presence of the AV up to the completion of virus cycle (24 h.p.i.) when total virus was titrated on Vero cell monolayers. Time 0 h.p.i. represents the end of virus adsorption (-2 to 0 h.p.i.). Dotted lines indicate the total virus production in the absence of inhibitor, corresponding to the value at 24 h.p.i.

To confirm the antiviral effect of CRL and VPA in ASFV infection, we analyzed the expression of virus proteins induced in Vero cells at late times of infection (Figure 4A). No late ASFV proteins (like p17 or p12) and a very low expression of the early virus protein p32 were detected in cultures infected in the presence of CRL or VPA, as reported in LG-treated ASFV-infected Vero cells (Hurtado et al., 2008). This decrease in protein synthesis was consistent with the inhibition observed in infective virus yield. The low levels of p32 detected, mainly at late times (24 h.p.i), are likely due to the accumulation of residual virus particles less sensitive to the AVs. Late ASFV protein P72 could not be quantified because of its co-migration with similar molecular weight-unspecific proteins, even detectable in mock samples.

We next studied the effect of the time of addition of the AVs on virus yield, assuming that assessing the step in virus cycle targeted by the drug may reveal information on the mechanism of action of the AV. As shown in Figure 4B, ASFV infection was greatly reduced when the AVs were added prior to virus adsorption. In the case of CRL, recovery of virus productivity was observed when CRL was added as early as 0 h after infection (simultaneous with the virus inoculum), which could support its implication in preventing virus attachment. Nevertheless, it has been described that CRL, among other inhibitors of cholesterol synthesis, affected ASFV production in Vero cells by inhibiting a fusion activity, without affecting the binding and internalization of the virus particles (Bernardes et al., 1998). While in that work, the inhibitor was added simultaneously to the virus, in our experiment, CRL was present 3 h before infection, allowing a possible effect of this compound on the cell membranes before virus adsorption.

On the other hand, the addition of VPA in successive times from 0 to 8 h.p.i. resulted in a gradual recovery of virus yields, and even a second infective step was noticed after 15 h.p.i.

Combined administration of different inhibitors is one of the strategies being currently explored to improve antiviral treatments (De Vleeschauwer et al., 2017). We studied the effect on ASFV infection of LG, VPA, and CRL, either alone or, searching for possible synergistic effects, in their possible combinations but most of the mixtures of two AVs resulted in an inhibitory effect similar to that obtained with the single drugs, or at least not higher than the sum of effects of both AVs. Nevertheless, in a few cases, combination of LG and VPA resulted in a slightly higher inhibitory effect than that expected from the sum of their individual effects (data not shown).

### Inhibition of Vesicular Porcine Viruses by Lauryl Gallate

We next analyzed the effect of LG in cells infected by different vesicular porcine viruses: FMDV, VSV, and SVDV. VPA was not included in this study since, as we previously reported, VSV is susceptible to this drug while FMDV and SVDV infections are not affected (Vazquez-Calvo et al., 2011). As shown in Figure 2, a consistent decrease of at least 1 log.u. in the production of both FMDV isolates from serotypes C (C-S8) and O (0-UKG 11/2001, named O-UK) in BHK21 or IBRS-2 cells at different M.O.I.s was observed in the presence of 10 μM LG (IC_50_: 0.2–2.5 μM at M.O.Is of 0.01–1 for C-S8; IC_50_: 0.7 μM at M.O.I of 1 for O-UK). In the case of cells infected with VSV or SVDV, the presence of LG reduced the virus productivity by 2 or 1 log.u., respectively (IC_50_: 0.9 μM for VSV; IC_50_: 1.7 μM for SVDV).

### Characterization of the Inhibition Exerted by Lauryl Gallate on Foot and Mouth Disease Virus Infection

**Figure 5:**
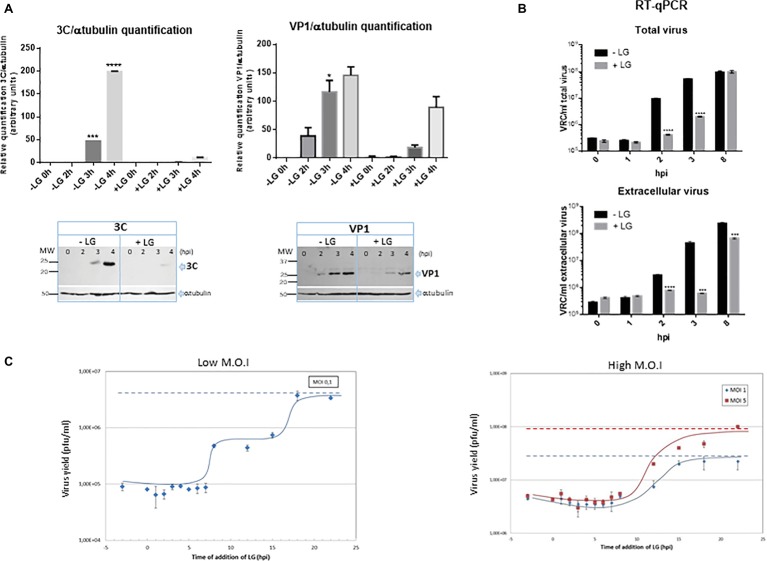
Inhibition exerted by LG on FMDV infection. **(A)** Protein expression. BHK21 cell monolayers were preincubated or not with 10 μM LG and infected with FMDV C-S8 isolate at a M.O.I of 2 pfu/cell. Non-adsorbed virus was washed away and the cultures maintained in the presence or absence of LG for 4 h. Cell extracts obtained at different h.p.i. were subjected to western blot analysis to detect FMDV structural (VP1) and non-structural (3C) proteins. FMDV-induced proteins are indicated by arrows. Molecular weight markers are shown (MW) in the left lane. Graphs show relative quantification of bands corresponding to VP1 and 3C proteins versus α-tubulin expression control. **(B)** RNA quantification. BHK21 cells were preincubated or not with 10 μM LG and infected with FMDV C-S8 isolate at a M.O.I of 2 pfu/cell. Non-adsorbed virus was washed away and the cultures maintained in the presence or absence of LG for 8 h. Total viral RNA (intracellular and extracellular) was extracted at different times p.i. and amplified by RT-qPCR. Data are expressed as VRC (number of viral RNA copies)/ml of total or extracellular virus. Detection limit was 10^4^ VRC/ml. **(C)** Effect of time of addition of LG on its inhibitory activity. Vero cells were infected with FMDV C-S8 isolate at a M.O.I. of 0.1 pfu/cell (low M.O.I.) or 1 or 5 pfu/cell (high M.O.I.), and 10 μM LG was added at different times before or after infection, maintaining the presence of LG up to 24 h.p.i. when total virus was titrated on BHK21 cell monolayers. Time 0 h.p.i. represents the end of virus adsorption (-2 to 0 h.p.i.). Statistically significant differences between control and LG-treated cells are indicated by asterisks (Student’s *t-*test, *p < 0.05, ***p < 0.001, ****p < 0.0001).

We further characterized the effect of LG on FMDV infection, as the availability of cost-effective antivirals for this virus, that severely impacts global livestock security, is limited (De Vleeschauwer et al., 2017). To determine the effect of LG on FMDV protein synthesis, the expression of structural (VP1) and non-structural (3C) FMDV proteins was analyzed at different times after virus infection in the presence or absence of the AV. As expected, the expression of both proteins was significantly reduced (Figure 5A), which is consistent with the level of inhibition (1 log.u.) detected in infective virus production. Likewise, LG transiently inhibited positive strand viral RNA synthesis, from 2 to 3 h.p.i., when total and extracellular viruses were analyzed (Figure 5B). At 8 h.p.i., the amount of intracellular FMDV RNA was not affected by LG, although extracellular virus was significantly reduced, which might reflect a decrease of infective virus release in the presence of LG. However, as shown in Figure 2, a consistent decrease of at least 1 log.u. was observed in the total virus production of two FMDV serotypes in cells infected at 24 h.p.i, and at three different M.O.I.s, supporting the inhibitory effect of LG at late times in FMDV infection.

The effect of time of addition of LG in FMDV infection revealed that virus productivity was blocked when LG was added up to 7 h.p.i. (Figure 5C), indicating that the step inhibited by the drug was late in infection (considering that the FMDV cycle is completed in about 8–10 h). With a M.O.I. of 0.1 pfu/cell, it is expected that two successive virus cycles have occurred at 24 h.p.i. The results in Figure 5C (“low M.O.I.”) are consistent with this model showing a plateau in virus titers from 8 to 15 h.p.i. (when the second virus round is inhibited by LG additions), with the total virus productivity recovered when LG was added at 20–24 h.p.i. A similar result was reported for the inhibitory action of LG on ASFV infection in Vero cells (Hurtado et al., 2008); in this case, virus production was inhibited when LG was added up to 5 h.p.i. and the maximum virus titer was observed from 15 h.p.i., involving the viral DNA replication as the LG-sensitive step in virus inhibition. As shown in Figure 5C (“high M.O.I.”), when higher M.O.I.s (1 or 5 pfu/cell) were used, most cells resulted initially FMDV-infected leading to a single round of infection.

### Effect of Esters of Gallic Acid Other Than Lauryl Gallate

**Figure 6:**
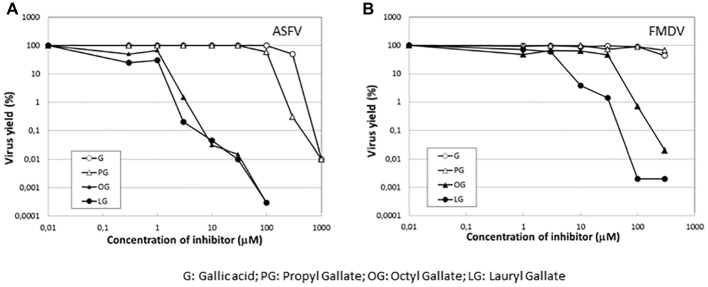
Specificity of the inhibition exerted by LG on FMDV infection. Vero cell monolayers for ASFV or BHK21 for FMDV were incubated for 1 h with G, PG, OG, or LG at the indicated concentrations (0–1,000 μM) and then infected with ASFV Ba71V strain or FMDV C-S8 strain in medium containing the same gallate esters concentrations. Total virus produced at 24 h.p.i. was titrated by plaque assay on BHK21 (FMDV) or Vero (ASFV) cell monolayers in duplicate samples and represented as virus yield (%).

To determine whether the inhibition of virus infection was also observed with esters of gallic acid other than LG, the effect of gallic acid (G) and its esters, propyl gallate (PG), octyl gallate (OG) (Uozaki et al., 2007; Yamasaki et al., 2007), and LG, was analyzed in FMDV-infected BHK21 cells. In this experiment, ASFV-infected Vero cells were also included. As represented in Figure 6, LG was the only compound that inhibited FMDV infection, reducing virus yield by up to 2 log.u, in a range of concentrations (10–30 μM) that still maintained cell viability. Neither G nor PG inhibited FMDV growth at non-toxic concentrations (up to 100 μM) while OG reduced virus yields by 2 log.u. at 100 μM, an effect that could be due, at least in part, to the cell toxicity exerted by this OG concentration. This result contrasts with the inhibitory effect of OG observed in ASFV (Figure 6), supporting that different viruses can show distinct sensitivities to an antiviral targeted against cellular functions, likely depending on their requirement on the affected cell function.

### “*In vivo*” Model for Foot and Mouth Disease Virus Inhibition by Lauryl Gallate

**Figure 7:**
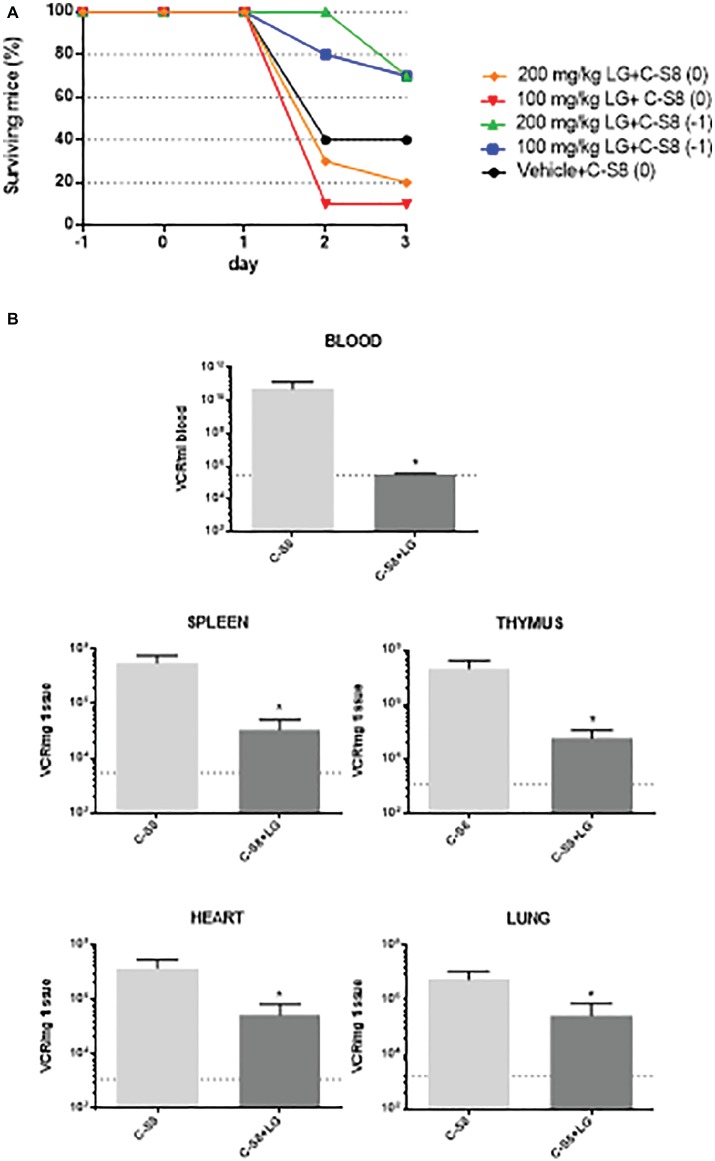
*In vivo* model for FMDV inhibition by LG. **(A)** Survival curves of C57 mice (*n* = 10) injected i.p. with a dose of 100 mg/kg or 200 mg/kg of LG once daily for 3 (group 0) or 4 (group −1) consecutive days. Mice were challenged with 1,000 pfu of FMDV C-S8 following 1 h (groups 0) or 24 h (group −1) after administration of LG, and were monitored daily for survival rate and clinical score. **(B)** RNA quantification. At day 2 p.i., three mice from the two groups that were inoculated or not, 24 h previous to infection (−1) with 100 mg/ml of LG, were bled and euthanized. RNA was extracted from blood, spleen, thymus, heart, and lung and quantified by RT-qPCR. Statistically significant differences in the number of VRC (viral RNA copies) per ml of blood or per mg of tissue between control and LG-treated animals are indicated by asterisks (Student’s *t-*test, *p < 0.05). Detection limit is indicated as a discontinuous line.

C57 mice were chosen for *in vivo* studies, as this mouse strain is clinically susceptible for FMDV infection (Salguero et al., 2005). A preliminary assay for LG toxicity in C57 mice revealed non-toxic working concentrations of up to 200 mg/kg administered twice daily for 5 consecutive days (data not shown). We next evaluated the protective efficacy of LG against intraperitoneal challenge of FMDV C-S8 isolate in these mice (*n* = 10 per group). A dose of 100 mg/kg or 200 mg/kg of LG was administered intraperitoneally once daily for 3 (group 0) or 4 (group −1) consecutive days. Mice were challenged with 1,000 pfu of FMDV C-S8 following 1 h (group 0) or 24 h (group −1) after administration of LG and animals were monitored daily for survival rate and clinical score. As reported, FMDV produced a limited mortality in C57 mice, with a survival rate of 40% in LG-untreated virus-infected controls (Figure 7A). Simultaneous inoculation of LG and FMDV conferred no protection (group 0), while inoculation of LG 24 h prior to viral challenge (group −1) increased survival rate to 70% at LG doses of 100 mg/kg and 200 mg/kg. Although both doses conferred similar protection, mice treated with 200 mg/kg showed lower weights than those treated with 100 mg/kg dose.

Next, we studied whether LG treatment reduced the virus load upon mice infection. To this end, a new experiment was performed using the protocol (−1), that is, LG injection 24 h previous to infection, with a dose of 100 mg/ml of LG administered intraperitoneally for 4 consecutive days. Animals were bled and euthanized for further dissection at day 2 p.i. Total (intracellular and extracellular) virus RNA was extracted from blood, spleen, lung, heart, and thymus samples. FMDV cDNA was synthesized using reverse transcriptase, and qPCR was performed. As shown in Figure 7B, a significant reduction of viral RNA copies (VRC) was found in all samples from FMDV-infected C57 mice that had received one daily dose of LG (100 mg/kg) starting 24 h before virus inoculation.

Overall, these results support that LG can also exert an *in vivo* antiviral effect, in a mouse model, reducing FMDV mortality and viral load in C57 mice when administered 24 h previous to infection and during the infection process.

Our results provide further characterization of the antiviral effect in cultured cells of the inexpensive drugs VPA and LG against a highly relevant animal virus such as ASFV that is quickly spreading in regions of Europe and Asia. The results also extend the antiviral potential of LG to FMDV and SVDV causing vesicular diseases in animals. LG, but not other esters of gallic acid, can significantly reduce the FMDV growth in cultured cells by inhibiting a late step in FMDV infection. Interestingly, LG also reduced the mice mortality upon infection with FMDV, which causes an economically devastating livestock disease. To our knowledge, this is one of the first descriptions of a cell-targeted compound with antiviral effect *in vivo* against FMDV with potential for cost-affordable antiviral intervention.

These findings reinforce the interest of testing the effect of these compounds in natural hosts as a first line of disease defense in case of outbreaks and as a vaccine complement to minimize the gap in antibody-mediated protection evoked by FMD vaccines.

## Data Availability

All datasets generated for this study are included in the manuscript and/or the supplementary files.

## Ethics Statement

All the experiments with infectious viruses were conducted in biosafety level 3 facilities and approved by the Ethical Committee of Animal Experimentation of Instituto Nacional de Investigación y Tecnología Agraria y Alimentaria (INIA, Madrid, Spain) and by the division of Animal Protection of the Comunidad de Madrid (PROEX 214/15). Animals were handled in strict accordance with the guidelines of European Community 86/609/CEE.

## Author Contributions

PL, FS, and AC conceived and designed the experiments. PL, MB, ET, and RC-A performed the experiments. PL, ET, FS, and AC analyzed the data and wrote the manuscript. All authors contributed to manuscript revision, read and approved the submitted version.

### Conflict of Interest Statement

The authors declare that the research was conducted in the absence of any commercial or financial relationships that could be construed as a potential conflict of interest.
